# Breast density change as a predictive surrogate for response to adjuvant endocrine therapy in hormone receptor positive breast cancer

**DOI:** 10.1186/bcr3221

**Published:** 2012-07-06

**Authors:** Jisun Kim, Wonshik Han, Hyeong-Gon Moon, Soo Kyung Ahn, Hee-Chul Shin, Jee-Man You, Sae-Won Han, Seock-Ah Im, Tae-You Kim, Hye Ryoung Koo, Jung Min Chang, Nariya Cho, Woo Kyung Moon, Dong-Young Noh

**Affiliations:** 1 Department of Surgery, Seoul National University College of Medicine, 101 Daehakro, Seoul, 110-744, Korea; 2 Cancer Research Institute, Seoul National University, 101 Daehakro, Seoul, 110-744, Korea; 3 Department of Surgery, Chung-Ang University College of Medicine, 102 Heuksukro, Seoul, 156-755, Korea; 4 Department of Surgery, Sun General Hospital, 29 Mokjungro, Daejeon, 301-725, Korea; 5 Department of Internal Medicine, Seoul National University College of Medicine, 101 Daehakro, Seoul, 110-744, Korea; 6 Department of Radiology, Seoul National University College of Medicine, 101 Daehakro, Seoul, 110-744, Korea

## Abstract

**Introduction:**

Anti-estrogen therapy has been shown to reduce mammographic breast density (MD). We hypothesized that a short-term change in breast density may be a surrogate biomarker predicting response to adjuvant endocrine therapy (ET) in breast cancer.

**Methods:**

We analyzed data for 1,065 estrogen receptor (ER)-positive breast cancer patients who underwent surgery between 2003 and 2006 and received at least 2 years of ET, including tamoxifen and aromatase inhibitors. MD was measured using Cumulus software 4.0 and expressed as a percentage. MD reduction (MDR) was defined as the absolute difference in MD of mammograms taken preoperatively and 8-20 months after the start of ET.

**Results:**

At a median follow-up of 68.8 months, the overall breast cancer recurrence rate was 7.5% (80/1065). Mean MDR was 5.9% (range, -17.2% to 36.9%). Logistic regression analysis showed that age < 50 years, high preoperative MD, and long interval between start of ET to follow-up mammogram were significantly associated with larger MDR (p < 0.05). In a survival analysis, tumor size, lymph node positivity, high Ki-67 (≥ 10%), and low MDR were independent factors significantly associated with recurrence-free survival (p < 0.05). Compared with the group showing the greatest MDR (≥ 10%), the hazard ratios for MDRs of 5-10%, 0-5%, and < 0% were 1.33, 1.92, and 2.26, respectively.

**Conclusions:**

MD change during short-term use of adjuvant ET was a significant predictor of long-term recurrence in women with ER-positive breast cancer. Effective treatment strategies are urgently needed in patients with low MDR despite about 1 year of ET.

## Introduction

Adjuvant endocrine therapy is the most effective systemic treatment modality for patients with hormone receptor (ER)-positive breast cancer, although many patients experience tumor recurrence during or after completion of endocrine therapy. Identifying factors that can predict disease recurrence early during adjuvant treatment may result in a more tailored strategy for patients likely to be endocrine resistant and may improve their overall outcomes.

Mammographic breast density (MD) is defined by the relative proportion of radiopaque areas, indicating the presence of fibroglandular tissue among the surrounding fatty component of the breast. High MD is associated with increased risk of breast cancer in both Western and Asian women [1,2]. The degree of lobular involution is known to have inverse correlation with breast cancer risk as well [3].

Studies on the efficacy of tamoxifen for chemoprevention of breast cancer in high-risk women have shown that MD is decreased following tamoxifen treatment [4,5]. Moreover, 12- to 18-month change in MD was found to be an excellent predictor of response to tamoxifen in the preventive setting [5]. However, no studies to date have addressed the association between MD reduction and the efficacy of adjuvant endocrine treatment in breast cancer patients. Using quantitative imaging analysis software to assess serial changes in MD, we investigated the association between the degree of MD reduction and long-term breast cancer recurrence in ER-positive breast cancer patients who received adjuvant endocrine therapy.

## Materials and methods

### Study population

Using our institution's prospectively maintained web-based database, we identified a total of 1,542 ER-positive breast cancer patients who underwent curative surgery at Seoul National University Hospital between October 2003 and December 2006. Patients were excluded if: 1) they did not receive adjuvant endocrine treatment, such as tamoxifen or an aromatase inhibitor, or were treated for less than 2 years; 2) their digital mammogram images were not available; 3) they had bilateral breast cancer, or 4) distant metastasis was observed before the start of endocrine therapy. Clinical and pathologic information on the 1,065 subjects was obtained from the database and used for further analysis. Treatment with adjuvant chemotherapy and/or radiotherapy was generally decided according to the institution's guidelines. The standard duration of treatment with tamoxifen is 5 years. Postmenopausal women were treated with the aromatase inhibitors anastrozole and letrozole for up to 5 years after surgery or after 2 to 3 years of tamoxifen.

### Mammographic density measurement

MD was quantitatively measured on cranio-caudal (CC) images of the unaffected breast using Cumulus software 4.0 (University of Toronto, Toronto, Canada) by a single investigator (JK) blinded to treatment outcome. All evaluated images were digital mammograms performed at our institution, so film scanning was unnecessary. Mammographic density reduction (MDR) was based on two digital mammograms; the first was taken within 2 weeks pre-surgery (preMD), and the second 8 to 20 months after the start of adjuvant endocrine treatment (postMD), and defined as the absolute difference between the MD of these two images (% MDR = % preMD - % postMD). The MD reduction ratio (MDRR) was also calculated (% MDRR = (preMD -postMD) × 100/preMD). Intraobserver reproducibility, tested for 10% of randomly selected images (213/2,130), was 0.93 (Pearson correlation coefficient).

### Statistical analysis

Change in MD was categorized into four levels, an increase (MDR < 0), 0 ≤ MDR < 5%, 5 ≤ MDR < 10%, and MDR ≥ 10%, and into a binary variable (MDR ≥ 5% and < 5%), with the 5% and 10% absolute reduction cut-offs based on previous findings [5]. We also analyzed absolute MDR as a continuous variable. The chi-square test and *t*-test were used to compare factors that could affect change in MD.

All loco-regional or distant disease recurrences were regarded as recurrence events in recurrence-free survival analysis. Survival curves were estimated by the Kaplan-Meier method and compared using the log-rank test. Multivariate analyses were conducted using Cox's proportional hazard regression model. All statistical analyses were performed using SPSS (version 17.0) software package (Chicago, IL, USA) and factors with *P *< 0.05 were considered statistically significant. Written informed consent was taken prior to surgery in all patients and the study protocol including the use of the database was approved by the Institutional Review Board of Seoul National University Hospital and met the guidelines of the responsible governmental agencies.

## Results

### Demographics and result of mammographic density measurement

The mean age of the 1,065 included patients was 49.1 years (range, 24 to 77 years) (Table 1), and their mean duration of overall endocrine therapy was 5.1 years (range, 0.9 to 7.9 years). One hundred and twenty-seven patients (11.9%) had ductal carcinoma *in situ *(DCIS). Second mammograms used for density measurements were taken an average 13.1 months (range, 8 to 20 months) after the start of endocrine therapy. The result of MD measurements (preMD, postMD, MDR, and MDRR) are shown in Table 2. Mean MDR was 5.9% (range, -17.2% to 36.9%).

**Table 1:** Patient demographics

Variable	Mean ± SD (range)	Number	%
Age, yr	49.0 ± 9.3 (24-77)		
≤ 50		680	63.8
> 50		385	36.2
Duration of ET, yr	5.0 ± 1.0 (0.9-7.9)		
ET regimen			
Tamoxifen 5 yr		657	61.7
Tamoxifen 2-3 yr - > AI (total 5 yr) yr		41	3.8
Tamoxifen 5 yr - > AI		192	18
AI 5 yr			16.4
Tumor size (cm)	2.1 ± 1.4 (0.1-10.0)		
≤ 2 cm		638	59.9
> 2 cm		427	40.1
Lymph node status			
Negative		706	66.3
Positive		359	33.7
Histologic grade			
Low/Intermediate		825	77.5
High		240	22.5
Progesterone receptor			
Negative		477	44.8
Positive		588	55.2
HER2			
Negative		976	91.6
Positive		88	8.3
Ki-67			
< 10		895	84
≥ 10		169	15.9
Neoadjuvant chemotherapy			
No		1017	95.5
Yes		48	4.5
Operation			
Breast conserving surgery		667	62.6
Mastectomy		398	37.4
Adjuvant chemotherapy			
No		247	23.2
Yes		818	76.8
Radiotherapy			
No		408	38.3
Yes		657	61.7
Recurrence			
Total		80	7.5
Locoregional		21	26.2
Contralateral breast		8	10
Distant metastasis		51	63.8

Clinicopathological factors of the 1065 patients. ET: endocrine therapy; AI: aromatase inhibitor; HER2: Human Epidermal Growth Factor Receptor 2.

**Table 2:** Distribution of mammographic density before and after treatment and change in mammographic density

Variable	Mean (range)	Number	%
PreMD, %*	35.77 ± 13.94 (5.42-82.18)		
< 10%		26	2.4
10% - 25%		223	20.9
25% - 50%		641	60.2
≥ 50%		175	16.4
			
PostMD, %	29.84 ± 12.12 (3.90-72.31)		
< 10%		35	3.3
10% - 25%		364	34.2
25% - 50%		611	57.4
≥ 50%		55	5.2
			
MDR, %	5.92 ± 7.08 (-17.2-36.9)		
< 5%		505	47.4
≥ 5%		560	52.6
< 0% (increased)		190	17.8
0%-5%		314	29.5
5%-10%		276	25.9
≥ 10%		285	26.8
			
MDRR, %			
< 15%		486	45.6
≥ 15%		579	54.4
< 0% (increased)		190	17.8
0%-10%		198	18.6
10%-25%		356	33.4
≥ 25%		321	30.1

Mammographic density reduction (MDR) and MDR ratio (MDRR) ((preMD - postMD) × 100/preMD) were initially evaluated as continuous variables then as dichotomized, quartered variables. Patients were divided into two groups (MDR < 5%, 5% ≤ MDR), and four groups (MDR ≥ 10%, 5% ≤ MDR < 10%, 0 ≤ MDR < 5%, and MDR < 0% (increased MD)). PreMD: initial preoperative mammographic density; PostMD: density of follow up mammography after 8 to 20 months of hormone therapy.

### Factors associated with density change

Patients were dichotomized by degree of MD reduction using a cut-off of 5% (MDR ≥ 5% vs. < 5%), and factors in the two groups were compared to identify associations with high MDR. Patients with MDR ≥ 5% were significantly younger (46.5 ± 8.0 vs. 51.9 ± 9.8 years, *P *< 0.001; Additional file 1, Table S1) and were significantly more likely to have been treated with tamoxifen than with an aromatase inhibitor (*P *< 0.001). Similarly, when MDR was analyzed as a continuous variable, mean MDR was higher in patients who received tamoxifen than in those who received an aromatase inhibitor (6.5 ± 7.1 vs. 3.1 ± 6.3%, *P *< 0.001, data not shown). Mean time from start of endocrine therapy to the second mammogram was longer in the group with MDR ≥ 5% than in the group with MDR < 5% (13.5 ± 3.1 vs. 12.6 ± 3.2 months, *P *< 0.001). Moreover, mean preMD was higher with MDR ≥ 5% than with MDR < 5% (40.9 ± 12.4% vs. 30.0 ± 13.3%, *P *< 0.001).

Multivariate logistic regression analysis was performed to identify factors independently associated with high MDR (Table 3). We found that age < 50 years, high preoperative MD, and long interval between start of endocrine therapy and the second mammogram were significantly associated with high MDR (*P *< 0.05). The data were consistently significant on stepwise regression analysis when adjusted by age, preMD and ET regimen (Additional file 1, Table S2).

**Table 3:** Factors associated with mammographic density reduction (MDR)

Variable	Odds ratio	95% Confidence interval	*P*-value
Age ≤ 50 yr	1.84	1.30, 2.61	0.001
Interval to follow-up mammography, months*	1.07	1.02, 1.12	0.006
Initial tamoxifen (vs. AI)	0.99	0.65, 1.51	0.958
PreMD, %	1.06	1.04, 1.07	< 0.001
Adjuvant chemotherapy	1.41	1.00, 2.00	0.052

Factors associated with mammographic density reduction (MDR). Odds ratios are shown for each factor having MDR < 5% (multivariate logistic regression analysis). *Interval between start of endocrine therapy and follow-up mammography. AI: aromatase inhibitor; PreMD: preoperative mammographic density.

### Density change and recurrence-free survival

**Figure 1:**
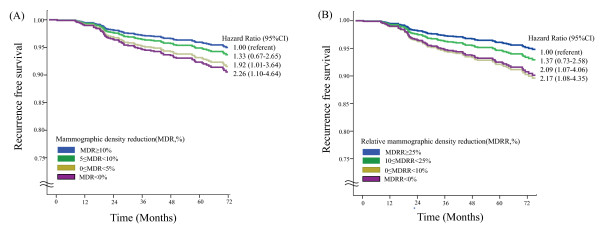
**Recurrence-free survival stratified by density reduction group**. (A) Recurrence-free survival according to mammographic density reduction (MDR). Patients were divided into four groups, MDR ≥ 10%, 5% ≤ MDR < 10%, 0 ≤ MDR < 5%, and MDR < 0% (increased MD). (B) Recurrence-free survival according to mammographic density reduction ratio (MDRR). Patients were divided into four groups, MDRR ≥ 25%, 10% ≤ MDRR < 25%, 0 ≤ MDRR < 10%, and MDRR < 0% (increased MDRR). Survival curves represent the results of Cox proportional hazard models adjusted for age, tumor size, lymph node, histologic grade, adjuvant chemotherapy, and Ki-67 status.

During a median follow-up of 67.7 months, 80 of the 1,065 patients (7.5%) experienced tumor recurrence (Table 1). Multivariate Cox regression analysis showed that when analyzed as a continuous variable, MDR was significantly associated with recurrence-free survival (hazard ratio (HR) = 0.95, 95% confidence interval (CI) 0.92 to 0.99, *P *= 0.005, Additional file 1, Table S3). When these patients were categorized into four groups according to the degree of MD change (the reference group with MDR ≥ 10%, plus three groups with MDR of 5 to 10%, 0 to 5%, and < 0% respectively), the HR for recurrence was proportional to the decrease in MDR. Compared with the reference group with the greatest MDR reduction (≥ 10%), the HRs for 5 to 10%, 0 to 5%, and < 0% MDR were 1.33 (*P *= 0.413), 1.92 (*P *= 0.048), and 2.26 (*P *= 0.027), respectively (Table 4, Figure 1A). Large tumor size, lymph node metastasis, and high Ki-67 level (≥ 10%) were also significantly associated with recurrence-free survival. The risk of recurrence was 1.67 times higher with a lower MDR of < 5% compared to MDR ≥ 5% when adjusted for age and preMD by forward selection stepwise analysis (HR 1.67, 95% CI 1.07 to 2.62, *P *= 0.025) (Additional file 1, Table S4).

**Table 4:** Predictive impact of mammographic density reduction (MDR) on recurrence-free survival

Variable	Hazard ratio	95% Confidence interval	*P*-value
Age, yr (continuous)	0.99	0.96, 1.02	0.393
MDR			
≥ 10% (reference)	1.00		0.101
5-10%	1.33	0.67, 2.65	0.413
0-5%	1.92	1.01, 3.64	0.048
< 0% (increased)	2.26	1.10, 4.64	0.027
Size, cm (continuous)	1.19	1.05, 1.35	0.006
Lymph node positive	2.02	1.20, 3.40	0.008
High histologic grade	1.29	0.78, 2.16	0.323
Chemotherapy	0.79	0.39, 1.60	0.520
Ki-67 ≥ 10%	1.77	1.05, 3.00	0.033

Cox proportional hazard regression model for recurrence-free survival. Risk of recurrence was analyzed using the group with the greatest reduction (MDR ≥ 10%) as the reference. MDR: absolute mammographic density reduction (pre-treatment - post-treatment).

**Figure 2:**
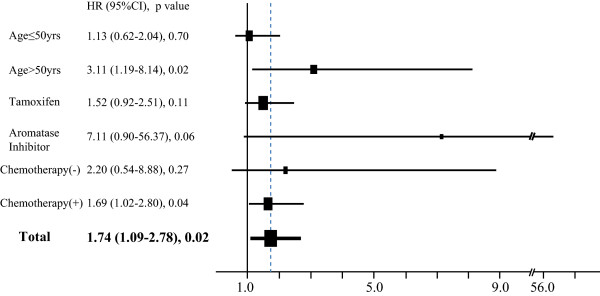
**Subgroup analysis of association between mammographic density reduction (MDR) and disease recurrence**. Forest plot shows hazard ratios (HRs) for recurrence in patients with MDR < 5% vs. those with MDR ≥ 5% in the different patient subgroups.

HRs for recurrence according to dichotomized MDR (≥ 5% vs. < 5%) in various subgroups are shown in a forest plot (Figure 2). Subgroup analysis showed that the association between low MDR and high risk of recurrence differed by age group (> 50 vs. ≤ 50 years), with MDR significantly associated with risk of recurrence only in the postmenopausal group. MDR also significantly predicted recurrence in patients taking AIs, and was strongly correlated with the factor of age. When adjusted by age and ET regimen the findings were consistent, showing low MDR as a significant risk factor for recurrence in patients who had undergone chemotherapy (HR 1.70, 95% CI 1.04 to 2.77, *P *= 0.033).

We also calculated the MDRR in this group of patients. We found that MDRR was significantly associated with risk of recurrence when analyzed as a continuous and as a binary variable. Compared with patients with MDRR ≥ 25%, the HRs for recurrence for patients with MDRR of 0 to 10% and MDRR < 0% were 2.09 and 2.17, respectively (*P *< 0.05 each; Table 5, Figure 1B). After adjusting for confounding factors, patients with MDRR ≥ 15% had a higher risk of recurrence than patients with MDRR < 15% (HR 1.60, 95% CI 1.02 to 2.50, *P *= 0.041) (Additional file 1, Table S5).

**Table 5:** Predictive impact of mammographic density reduction ratio (MDRR) on recurrence-free survival

Variable	Hazard ratio	95% Confidence interval	*P*-value
Age, yr (continuous)	0.99	0.97-1.02	0.600
MDRR (%)*			
≥ 25%	1.00	Referent	0.078
10%-25%	1.37	0.73-2.58	0.327
0%-10%	2.09	1.07-4.06	0.030
< 0% (increased)	2.17	1.08-4.35	0.030
Size, cm (continuous)	1.19	1.05-1.35	0.007
Lymph node positive	1.98	1.18-3.34	0.010
High histologic grade	1.34	0.80-2.24	0.261
Chemotherapy done	0.79	0.39-1.60	0.519
Ki-67 ≥ 10%	0.60	0.33-0.94	0.029

Cox proportional hazard regression model for recurrence-free survival. Risk of recurrence was analyzed using the group with the greatest reduction (MDRR ≥ 25%) as the reference. *MDRR = (pre-treatment MD - post-treatment MD) × 100/pre-treatment MD.

## Discussion

We have shown here that short-term MDR is predictive of long-term outcomes following endocrine therapy in patients with ER-positive breast cancer. Patients who experienced < 5% absolute MDR and those with increased MD after about 1 year of endocrine therapy were at 1.92- and 2.26-fold greater risk of recurrence respectively, than patients with MDR ≥ 10%. This association was also observed when absolute MDR was analyzed as a continuous variable, and when MDRR rather than absolute MDR was assessed.

Regardless of the evolution of anti-estrogen therapy, a substantial proportion of patients with ER-positive breast cancer experience disease recurrence during follow-up. Effective biomarkers are needed to predict endocrine resistance despite ER expression. Many previous investigations have focused on tumor factors associated with endocrine resistance [6,7]. The level of MDR resulting from endocrine treatment is a host factor indicating individual susceptibility to endocrine agents. These findings support the hypothesis that host response to adjuvant endocrine therapy may be as important and should be considered in addition to the clinicopathological characteristics of the primary tumor.

Breast density is one of the strongest risk factors for breast cancer development [8]. A recent study showed that the magnitude of the association of exogenous or endogenous hormone exposure and mammographic density change is related to future risk of breast cancer [9]. Cuzick *et al*. conducted a nested case-control study within the IBIS-I study, a randomized prevention trial of tamoxifen versus placebo to determine the association between tamoxifen-induced density change and breast cancer risk. They showed that the 12- to 18-month change in mammographic breast density is an excellent predictor of tamoxifen efficacy in the preventive setting [5].

Our findings raise the question of whether treatment strategy should be altered based on change in MD after only one year of endocrine therapy. For the clinical application of individualized therapy, studies are needed to evaluate the ability of shorter-term changes in MD, such as after < 6 months, to predict risk of recurrence, and new treatment strategies also should be tested according to the predicted result.

We also found that factors such as age < 50 years, high preoperative MD, and long interval between the start of endocrine therapy and the second mammogram were significantly associated with high MDR, indicating that a dense breast *per se *is not a sign of endocrine resistance, and that MD decreases more with prolonged endocrine treatment.

Although MDR was greater in patients < 50 years of age than in those aged ≥ 50 years, the degree of MDR was not associated with recurrence in younger patients. The reason for this is not clear, although it may be due to the complicated hormonal milieu and factors other than endocrine therapy affecting breast density in younger women. The relatively small number of events in patients aged < 50 years (43/680, 7.1%) in our dataset could have affected the statistical significance and further evaluation within a larger dataset is required.

The degree of MDR may differ according to the type of endocrine therapy. Tamoxifen was reported to be associated with an 8% mean absolute reduction in breast density at 1.5 years and a reduction of 14% after 4.5 years [10,11]. Raloxifene has been reported to decrease absolute breast density by 1.5% per year [12]. In a small study involving 54 patients, adjuvant anastrozole had no effect on breast density in the contralateral breast after 6 months and resulted in a 16% relative reduction (*P *= 0.08) after 12 months [13]. Letrozole and exemestane also did not reduce mammographic breast density [14]. Addition of an aromatase inhibitor to hormone replacement therapy resulted in a significant reduction in breast density [1]. In a univariate analysis we found that tamoxifen treatment was associated with a higher MDR than treatment with aromatase inhibitors (*P *< 0.001). However, an association between MDR and recurrence-free survival was observed in our aromatase inhibitor group.

There is no definite evidence-based mechanism for an association between anti-estrogen therapy, reduced breast density and a better outcome. With aromatase inhibitors, it is possible that reduced density reflects effective circulating estrogen deprivation, and as a result, also affects micro-metastases. Another explanation is the difference in the drug metabolism efficiency of the host. Patients with adequate serum drug concentrations should have a better response and outcome. However, the metabolic mechanism of tamoxifen and aromatase inhibitors must be different. Adherence to a prescribed drug can partly explain the correlation between density change and patient outcome. In premenopausal and perimenopausal women, a good chemotherapy response could cause chemotherapy-induced ovarian failure, and as a result, could reduce breast density and improve disease-free survival. It is unknown, but is less likely that tumor-associated fibroblasts or other stromal cells in the breast might directly affect distant micro-metastatic cancer cells.

We also found that 17.8% of our study subjects had increased MD after endocrine therapy. Similarly, in the preventive tamoxifen study IBIS-1, 11% of patients treated with tamoxifen and 24% given placebo had an increased MD. The mechanism of this increase in MD is unknown. Investigations are needed to determine whether in these women, tamoxifen acts as an estrogen agonist in the breast.

The reproducibility of MD measurement is important, and studies indicate good intraobserver reproducibility with correlation coefficients of 0.92 to 0.96 [15]. We used Cumulus software, which has been the most widely used for MD measurements in previous studies. Using digital mammographic images, we found that the Pearson correlation coefficient was 0.93.

The major limitation of the study was the absence of data on factors that may be closely associated with breast density, such as body mass index. Because this study was retrospective in design, the timing of follow-up mammography was not uniform, varying from 8 to 20 months after initiation of endocrine therapy. Another limitation was that the study subjects received heterogeneous adjuvant therapy regimens. We also could not determine the degree of ER expression in tumors, a potential major factor affecting resistance to endocrine treatment.

To our knowledge, this study was the first to assess the value of MD change as a predictive surrogate in breast cancer patients receiving adjuvant endocrine therapy. The positive result we obtained warrants larger-scale prospective studies. Basic research to identify the molecular pathways related to endocrine resistance and mammographic density is also needed.

## Conclusion

In conclusion, low MDR or increased MD during short-term endocrine therapy was independently associated with poor recurrence-free survival in patients with ER-positive breast cancer. Change in MD may be predictive of response to adjuvant endocrine therapy. Studies should be designed to investigate how to use this valuable information in routine clinical practice.

## Abbreviations

AI: aromatase inhibitor; CC: cranio-caudal; ER: estrogen receptor; DCIS: ductal carcinoma *in situ*; ET: endocrine therapy; MD: mammographic density; MDR: mammographic density reduction; preMD: initial preoperative mammographic density; postMD: mammographic density after endocrine therapy; MDRR: mammographic density reduction ratio.

## Competing interests

The authors declare that they have no competing interests.

## Authors' contributions

All the authors have made substantial contributions to conception and design, acquisition of data, or analysis and interpretation of data. HRK, JMC, NC, and WKM carried out analysis of the imaging profiles of each patient including the Cumulus density measurement. SWH, SAI, and TYK confirmed patients' outcomes of recurrence and the adequacy of endocrine therapy. SKA, HCS, and JMY directly participated in the whole process throughout the research and statistical analysis. HGM and DYN participated in the study design and helped to draft the manuscript. JK measured the percent mammographic density and performed the research. As corresponding author, WSH designed and coordinated the research and provided close guidance throughout the process. All authors read and approved the final manuscript. The authors have been involved in drafting the manuscript or revising it critically for important intellectual content and have all given final approval of the version to be published.

## Supplementary Material

Additional file 1 **Table S1: Univariate analysis for mammographic density reduction (MDR)**. Analysis of factors associated with MDR divided into two group (MDR < 5% vs. MDR ≥ 5%). Younger age, tamoxifen use, longer interval from initial endocrine therapy, higher PreMD, adjuvant chemotherapy were likely to have higher MDR (≥ 5%). **Table S2: **Stepwise regression analysis (forward selection) of factors for MDR* ≥ 5%. After adjusting for the confounding factors, age, interval to follow up, and preoperative mammographic density, adjuvant chemotherapy was not independently associated with MDR. **Table S3: **Cox proportional analysis for recurrence-free survival (RFS): MDR as a continuous variable. MDR analyzed as a continuous variable was an independent risk factor for recurrence, along with size, lymph node (LN) status, and Ki-67 level. **Table S4: **Cox proportional hazard regression (forward selection) analysis for RFS. After adjusting for confounding factors, patients with MDR < 5% had 1.67 times significantly higher risk of recurrence than the MDR ≥ 5% group. Tumor size, lymph node (LN) positivity, and Ki-67 (cut-off 10%) were independent prognostic factors as known. **Table S5: **Cox proportional hazard regression (forward selection) analysis for RFS. After adjusting for confounding factors, patients with MDRR < 15% had 1.60 times significantly higher risk of recurrence than the MDRR ≥ 15% group (*P *= 0.041). Tumor size, lymph node (LN) positivity, and Ki-67 (cut-off 10%) were independent prognostic factors as known. Click here for file
