# Supplementary material for: Breast density change as a predictive surrogate for response to adjuvant endocrine therapy in hormone receptor positive breast cancer
Source: Breast Cancer Res. 2012 Jul 6;14(4):R102. doi: 10.1186/bcr3221 (PMC3680951; doi:10.1186/bcr3221)
Supplement: Additional file 1 — Table S1: Univariate analysis for mammographic density reduction (MDR). Analysis of factors associated with MDR divided into two group (MDR < 5% vs. MDR ≥ 5%). Younger age, tamoxifen use, longer interval from initial endocrine therapy, higher PreMD, adjuvant chemotherapy were likely to have higher MDR (≥ 5%). Table S2: Stepwise regression analysis (forward selection) of factors for MDR* ≥ 5%. After adjusting for the confounding factors, age, interval to follow up, and preoperative mammographic density, adjuvant chemotherapy was not independently associated with MDR. Table S3: Cox proportional analysis for recurrence-free survival (RFS): MDR as a continuous variable. MDR analyzed as a continuous variable was an independent risk factor for recurrence, along with size, lymph node (LN) status, and Ki-67 level. Table S4: Cox proportional hazard regression (forward selection) analysis for RFS. After adjusting for confounding factors, patients with MDR < 5% had 1.67 times significantly higher risk of recurrence than the MDR ≥ 5% group. Tumor size, lymph node (LN) positivity, and Ki-67 (cut-off 10%) were independent prognostic factors as known. Table S5: Cox proportional hazard regression (forward selection) analysis for RFS. After adjusting for confounding factors, patients with MDRR < 15% had 1.60 times significantly higher risk of recurrence than the MDRR ≥ 15% group (P = 0.041). Tumor size, lymph node (LN) positivity, and Ki-67 (cut-off 10%) were independent prognostic factors as known. [file bcr3221-S1.DOCX]

Additional material files :

Additional file : Supplementary Tables 1-5.

Supplementary table 1.

Factors associated with mammographic density reduction (MDR): Univariate analysis

| Variables | Group | MDR<5% | MDR≥5% | p-value |
| --- | --- | --- | --- | --- |
|  |  | (N=505) (%) | (N=560) (%) |  |
| Age (yr) | Mean ± SD | 51.91±9.82 | 46.47±8.08 | <0.001 |
|  | ≤50 | 243 (35.7) | 437 (64.3) | <0.001 |
|  | >50 | 262 (68.1) | 123 (31.9) |  |
|  |  |  |  |  |
| Initial ET regimen* | Tamoxifen | 393 (44.2) | 497 (55.8) | <0.001 |
|  | AI | 112 (64) | 63 (36) |  |
|  |  |  |  |  |
| Interval to f/u mmg (months)† |  | 12.58±3.17 | 13.48±3.05 | <0.001 |
|  |  |  |  |  |
| PreMD (%)‡ | Mean ± SD | 30.03±13.34 | 40.93±12.38 | <0.001 |
|  | <10% | 25 (96.2) | 1 (3.8) | <0.001 |
|  | 10%-25% | 172 (77.1) | 51 (22.9) |  |
|  | 25%-50% | 269 (42) | 372 (58) |  |
|  | 50%≤ | 39 (22.3) | 136 (77.7) |  |
|  |  |  |  |  |
| PostMD (%)§ | Mean ± SD | 29.90±13.34 | 29.79±10.91 | 0.885 |
|  | <10% | 27 (77.1) | 8 (22.9) | <0.001 |
|  | 10%-25% | 172 (47.3) | 192 (52.7) |  |
|  | 25%-50% | 271 (44.4) | 340 (55.6) |  |
|  | 50%≤ | 35 (63.6) | 20 (36.4) |  |
|  |  |  |  |  |
| Adjuvant chemotherapy | No | 149 (60.3) | 98 (39.7) | <0.001 |
|  | Yes | 356 (43.5) | 462 (56.5) |  |

* ET= Endocrine therapy

† Interval between endocrine therapy start to the postoperative follow-up mammography

‡ PreMD=Initial preoperative mammographic density

§ PostMD =Density of follow up mammography after 8-20months of hormone therapy

Supplementary table 2.

Cox proportional hazard regression model for recurrence-free survival: mammographic density reduction (MDR^*^) analyzed as a continuous variable.

| Variable | Hazard ratio | 95% Confidence interval | p-value |
| --- | --- | --- | --- |
| Age (yr, continuous) | 0.99 | 0.96-1.01 | 0.320 |
| MDR (%, continuous)^*^ | 0.95 | 0.92-0.99 | 0.005 |
| Size (cm, continuous) | 1.20 | 1.05-1.36 | 0.006 |
| Lymph node positive | 2.04 | 1.21-3.43 | 0.007 |
| High histologic grade | 1.30 | 0.78-2.17 | 0.320 |
| Chemotherapy done | 0.79 | 0.39-1.59 | 0.520 |
| Ki-67 ≥10% | 1.78 | 1.06-3.01 | 0.030 |

^*^MDR = Absolute mammographic density reduction, PreMD-PostMD.

Supplementary table 3.

Stepwise regression analysis (forward selection) was performed for factors associated with MDR^*^≥5%

| Variable | Odds ratio | 95% Confidence interval | *p*-value |
| --- | --- | --- | --- |
| Age ≤50 yr | 1.83 | 1.35-2.49 | <0.001 |
| Interval to follow-up mammography (months) | 1.07 | 1.02-1.12 | 0.006 |
| PreMD^*^ (%) | 1.06 | 1.04-1.07 | <0.001 |
| Adjuvant chemotherapy done | 1.41 | 1.00-2.00 | 0.049 |

^*^ MDR = Absolute mammographic density reduction, PreMD-PostMD.

† PreMD=Initial preoperative mammographic density**.**

Supplementary table 4.

Cox proportional hazard regression (forward selection) model for recurrence-free survival analysis.

| Variable | Hazard ratio | 95% Confidence interval | *p*-value |
| --- | --- | --- | --- |
| MDR^*^ <5% | 1.67 | 1.07-2.63 | 0.025 |
| Size (cm, continuous) | 1.20 | 1.06-1.36 | 0.004 |
| Lymph node positive | 2.01 | 1.20-3.40 | 0.004 |
| Ki-67 ≥10% | 1.96 | 1.05-3.00 | 0.007 |

^*^ MDR = Absolute mammographic density reduction, PreMD-PostMD.

Supplementary table 5.

Cox proportional hazard regression (forward selection) model for recurrence-free survival analysis.

| Variable | Hazard ratio | 95% Confidence interval | p-value |
| --- | --- | --- | --- |
| MDRR^*^ <15% | 1.60 | 1.02-2.50 | 0.041 |
| Size (cm, continuous) | 1.20 | 1.06-1.35 | 0.007 |
| Lymph node positive | 1.98 | 1.26-3.20 | 0.010 |
| Ki-67 ≥10% | 0.60 | 1.22-3.24 | 0.029 |

^*^ MDRR = (PreMD-PostMD) x 100 / PreMD
